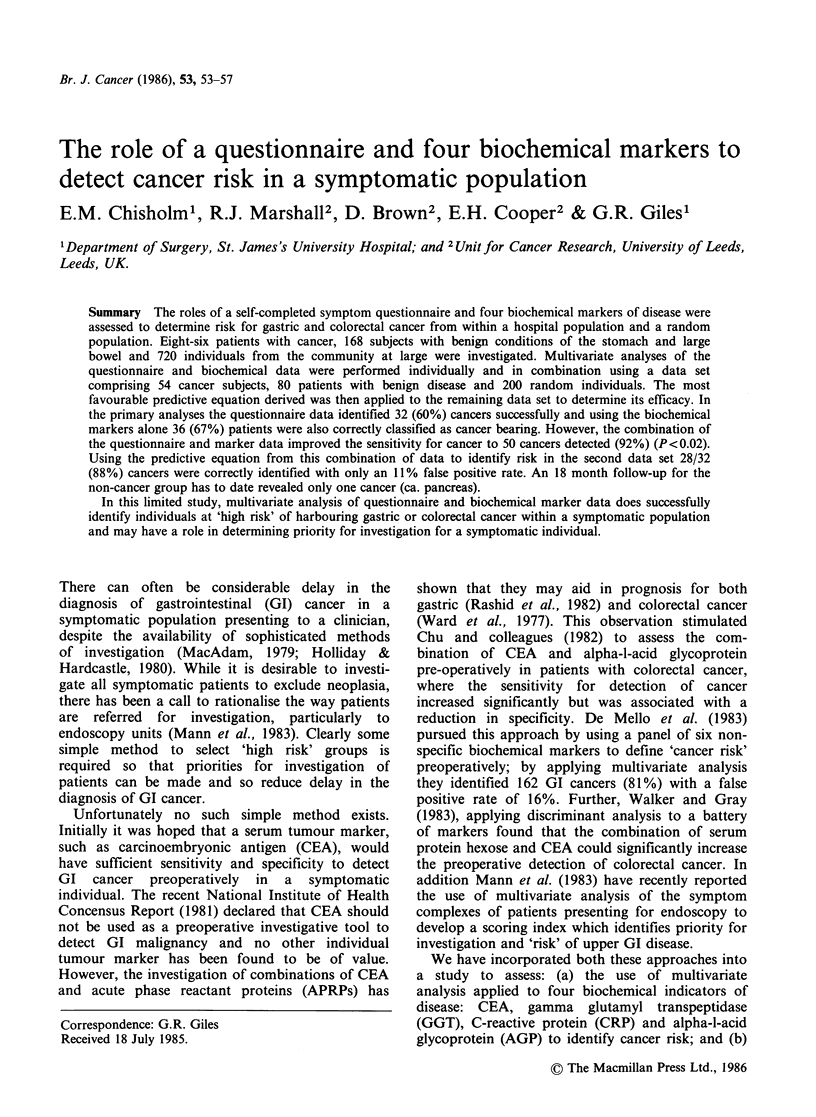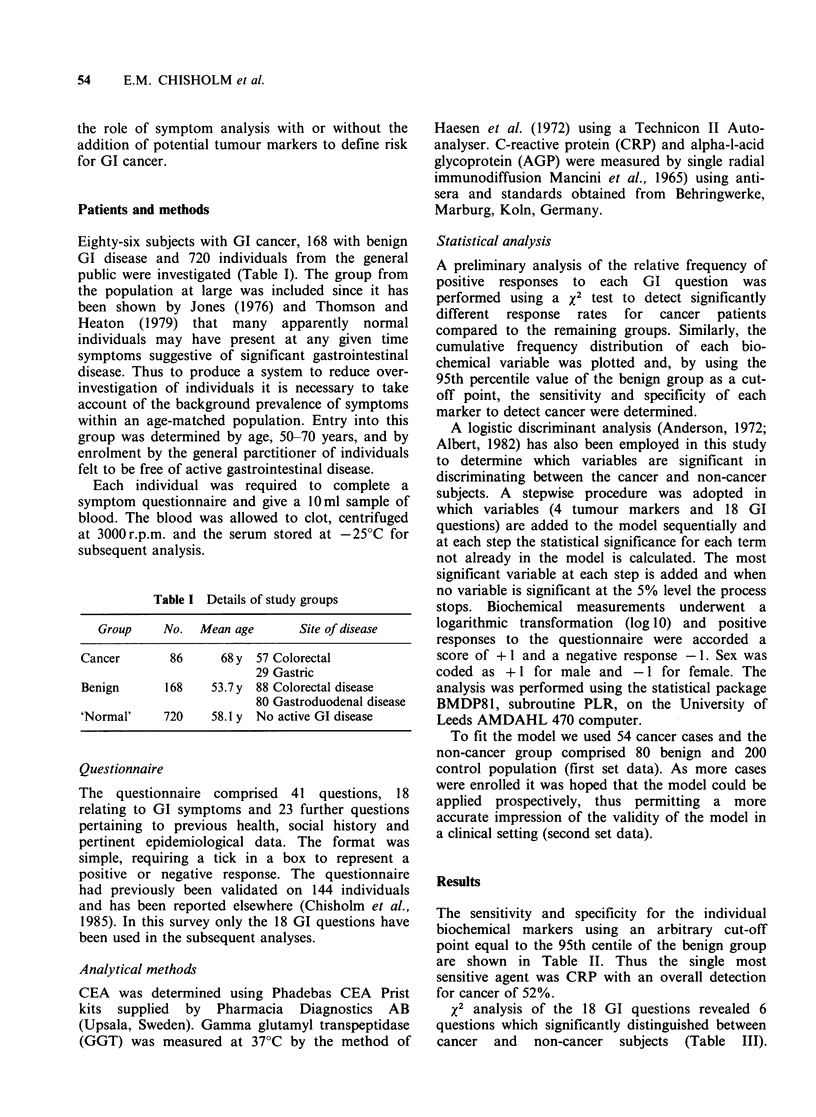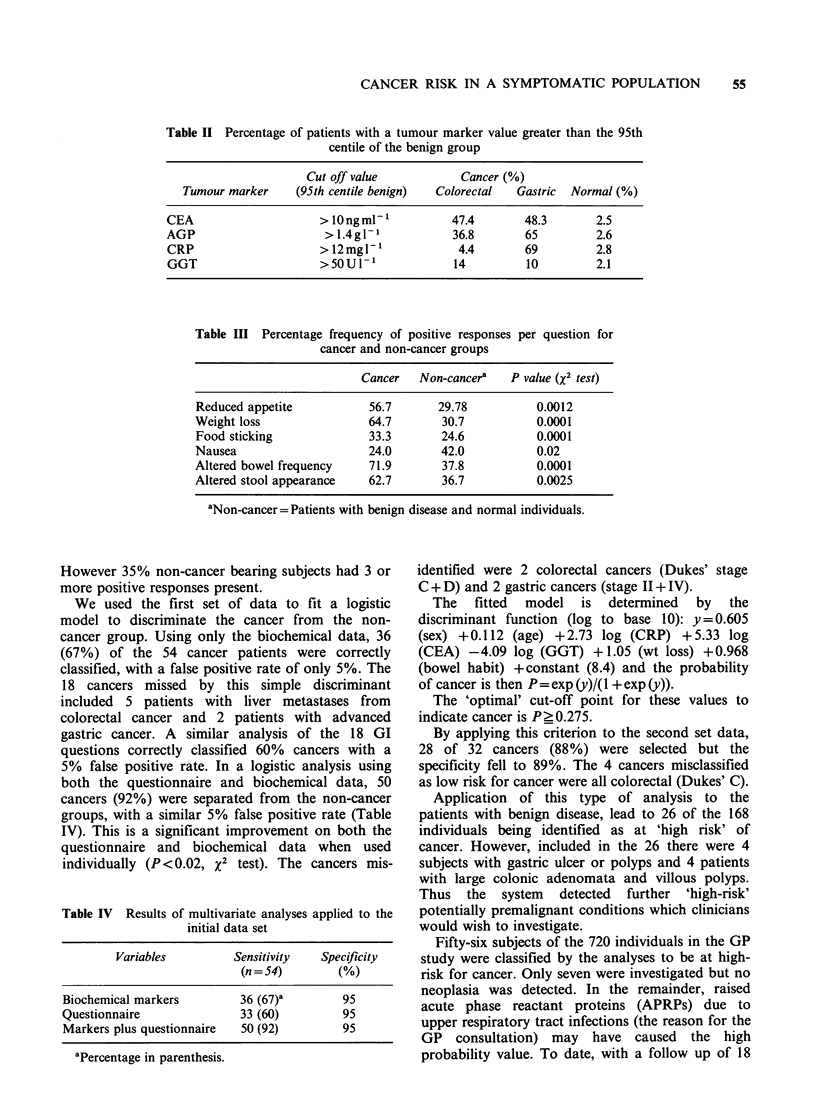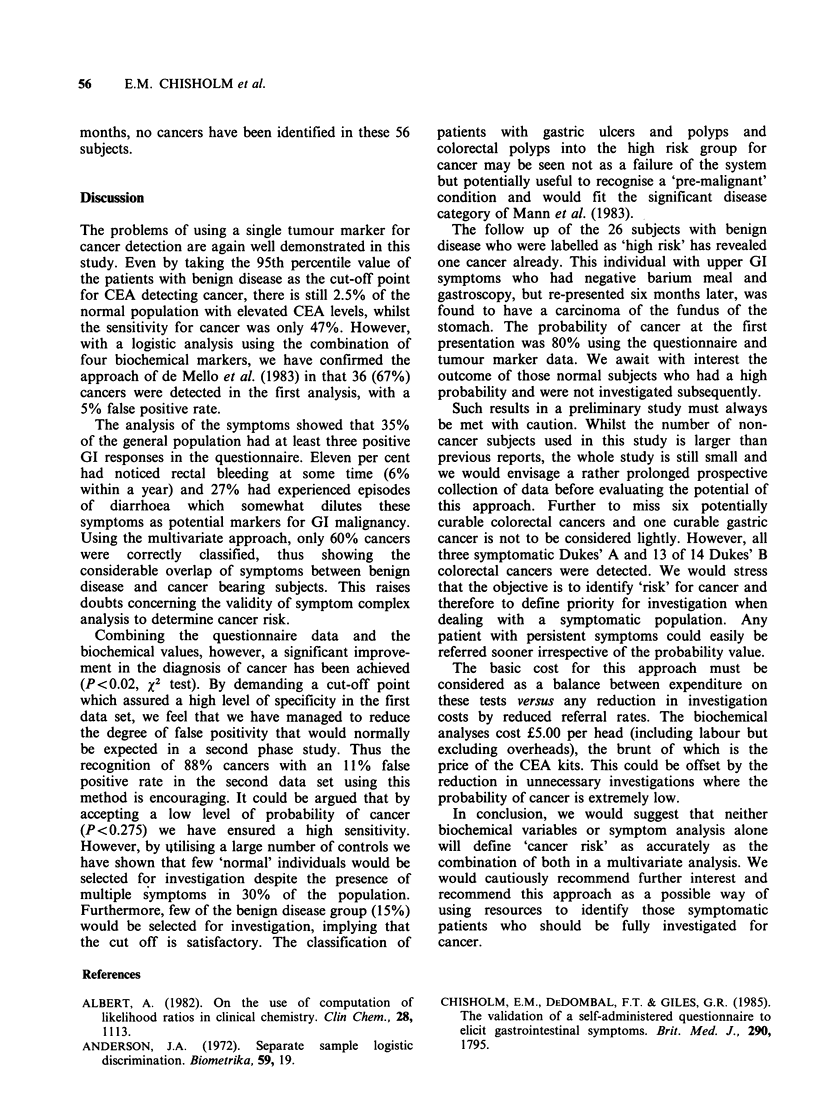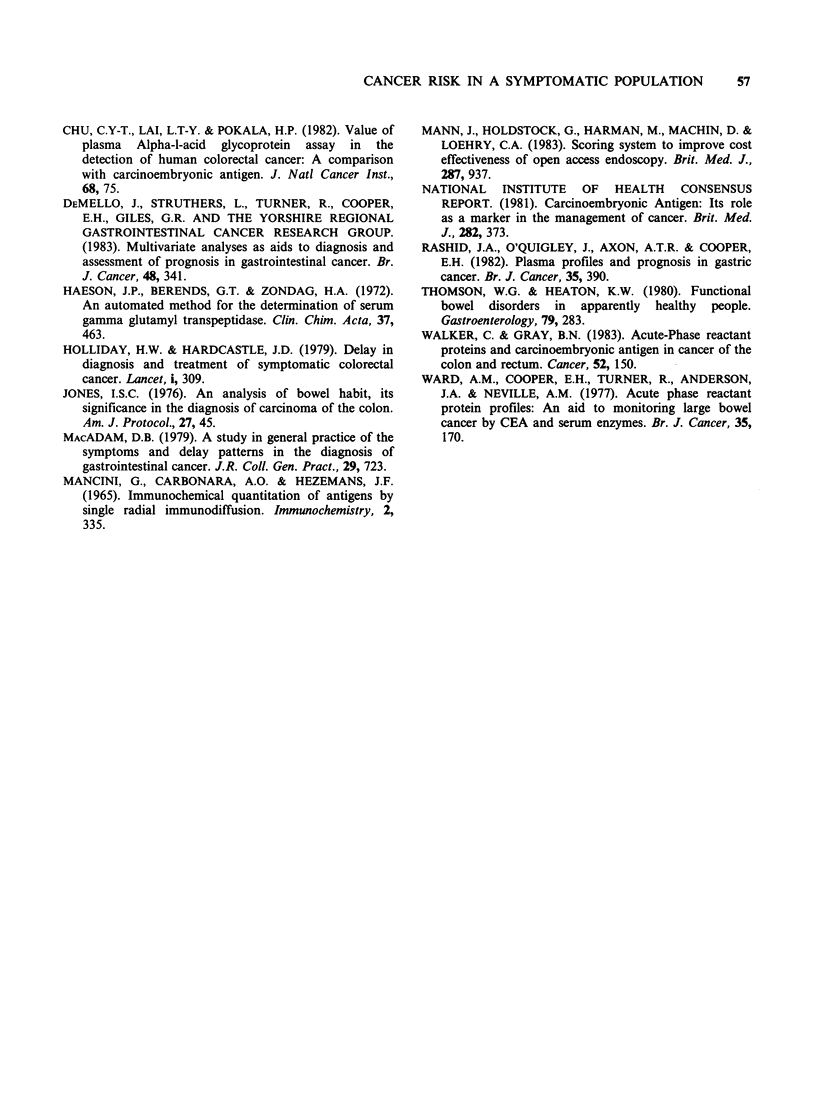# The role of a questionnaire and four biochemical markers to detect cancer risk in a symptomatic population.

**DOI:** 10.1038/bjc.1986.8

**Published:** 1986-01

**Authors:** E. M. Chisholm, R. J. Marshall, D. Brown, E. H. Cooper, G. R. Giles

## Abstract

The roles of a self-completed symptom questionnaire and four biochemical markers of disease were assessed to determine risk for gastric and colorectal cancer from within a hospital population and a random population. Eight-six patients with cancer, 168 subjects with benign conditions of the stomach and large bowel and 720 individuals from the community at large were investigated. Multivariate analyses of the questionnaire and biochemical data were performed individually and in combination using a data set comprising 54 cancer subjects, 80 patients with benign disease and 200 random individuals. The most favourable predictive equation derived was then applied to the remaining data set to determine its efficacy. In the primary analyses the questionnaire data identified 32 (60%) cancers successfully and using the biochemical markers alone 36 (67%) patients were also correctly classified as cancer bearing. However, the combination of the questionnaire and marker data improved the sensitivity for cancer to 50 cancers detected (92%) (P less than 0.02). Using the predictive equation from this combination of data to identify risk in the second data set 28/32 (88%) cancers were correctly identified with only an 11% false positive rate. An 18 month follow-up for the non-cancer group has to date revealed only one cancer (ca. pancreas). In this limited study, multivariate analysis of questionnaire and biochemical marker data does successfully identify individuals at "high risk' of harbouring gastric or colorectal cancer within a symptomatic population and may have a role in determining priority for investigation for a symptomatic individual.


*Br. J. Cancer* (1986), **53**, 53–57

### The role of a questionnaire and four biochemical markers to detect cancer risk in a symptomatic population

E.M. Chisholm[1], R.J. Marshall[2], D. Brown[2], E.H. Cooper[2] & G.R. Giles[1]

[1]*Department of Surgery, St. James's University Hospital; and* [2]*Unit for Cancer Research, University of Leeds, Leeds, UK.*

**Summary** The roles of a self-completed symptom questionnaire and four biochemical markers of disease were assessed to determine risk for gastric and colorectal cancer from within a hospital population and a random population. Eight-six patients with cancer, 168 subjects with benign conditions of the stomach and large bowel and 720 individuals from the community at large were investigated. Multivariate analyses of the questionnaire and biochemical data were performed individually and in combination using a data set comprising 54 cancer subjects, 80 patients with benign disease and 200 random individuals. The most favourable predictive equation derived was then applied to the remaining data set to determine its efficacy. In the primary analyses the questionnaire data identified 32 (60%) cancers successfully and using the biochemical markers alone 36 (67%) patients were also correctly classified as cancer bearing. However, the combination of the questionnaire and marker data improved the sensitivity for cancer to 50 cancers detected (92%) ($P<0.02$). Using the predictive equation from this combination of data to identify risk in the second data set 28/32 (88%) cancers were correctly identified with only an 11% false positive rate. An 18 month follow-up for the non-cancer group has to date revealed only one cancer (ca. pancreas).

In this limited study, multivariate analysis of questionnaire and biochemical marker data does successfully identify individuals at 'high risk' of harbouring gastric or colorectal cancer within a symptomatic population and may have a role in determining priority for investigation for a symptomatic individual.

There can often be considerable delay in the diagnosis of gastrointestinal (GI) cancer in a symptomatic population presenting to a clinician, despite the availability of sophisticated methods of investigation (MacAdam, 1979; Holliday & Hardcastle, 1980). While it is desirable to investigate all symptomatic patients to exclude neoplasia, there has been a call to rationalise the way patients are referred for investigation, particularly to endoscopy units (Mann *et al.*, 1983). Clearly some simple method to select 'high risk' groups is required so that priorities for investigation of patients can be made and so reduce delay in the diagnosis of GI cancer.

Unfortunately no such simple method exists. Initially it was hoped that a serum tumour marker, such as carcinoembryonic antigen (CEA), would have sufficient sensitivity and specificity to detect GI cancer preoperatively in a symptomatic individual. The recent National Institute of Health Concensus Report (1981) declared that CEA should not be used as a preoperative investigative tool to detect GI malignancy and no other individual tumour marker has been found to be of value. However, the investigation of combinations of CEA and acute phase reactant proteins (APRPs) has shown that they may aid in prognosis for both gastric (Rashid *et al.*, 1982) and colorectal cancer (Ward *et al.*, 1977). This observation stimulated Chu and colleagues (1982) to assess the combination of CEA and alpha-l-acid glycoprotein pre-operatively in patients with colorectal cancer, where the sensitivity for detection of cancer increased significantly but was associated with a reduction in specificity. De Mello *et al.* (1983) pursued this approach by using a panel of six non-specific biochemical markers to define 'cancer risk' preoperatively; by applying multivariate analysis they identified 162 GI cancers (81%) with a false positive rate of 16%. Further, Walker and Gray (1983), applying discriminant analysis to a battery of markers found that the combination of serum protein hexose and CEA could significantly increase the preoperative detection of colorectal cancer. In addition Mann *et al.* (1983) have recently reported the use of multivariate analysis of the symptom complexes of patients presenting for endoscopy to develop a scoring index which identifies priority for investigation and 'risk' of upper GI disease.

We have incorporated both these approaches into a study to assess: (a) the use of multivariate analysis applied to four biochemical indicators of disease: CEA, gamma glutamyl transpeptidase (GGT), C-reactive protein (CRP) and alpha-l-acid glycoprotein (AGP) to identify cancer risk; and (b)

Correspondence: G.R. Giles
Received 18 July 1985.

© The Macmillan Press Ltd., 1986

the role of symptom analysis with or without the addition of potential tumour markers to define risk for GI cancer.

### Patients and methods

Eighty-six subjects with GI cancer, 168 with benign GI disease and 720 individuals from the general public were investigated (Table I). The group from the population at large was included since it has been shown by Jones (1976) and Thomson and Heaton (1979) that many apparently normal individuals may have present at any given time symptoms suggestive of significant gastrointestinal disease. Thus to produce a system to reduce over-investigation of individuals it is necessary to take account of the background prevalence of symptoms within an age-matched population. Entry into this group was determined by age, 50–70 years, and by enrolment by the general parctitioner of individuals felt to be free of active gastrointestinal disease.

Each individual was required to complete a symptom questionnaire and give a 10 ml sample of blood. The blood was allowed to clot, centrifuged at 3000 r.p.m. and the serum stored at −25°C for subsequent analysis.

**Table I** Details of study groups

| *Group* | *No.* | *Mean age* | *Site of disease* |
|---|---|---|---|
| Cancer | 86 | 68 y | 57 Colorectal<br>29 Gastric |
| Benign | 168 | 53.7 y | 88 Colorectal disease<br>80 Gastroduodenal disease |
| 'Normal' | 720 | 58.1 y | No active GI disease |

### *Questionnaire*

The questionnaire comprised 41 questions, 18 relating to GI symptoms and 23 further questions pertaining to previous health, social history and pertinent epidemiological data. The format was simple, requiring a tick in a box to represent a positive or negative response. The questionnaire had previously been validated on 144 individuals and has been reported elsewhere (Chisholm *et al.*, 1985). In this survey only the 18 GI questions have been used in the subsequent analyses.

### *Analytical methods*

CEA was determined using Phadebas CEA Prist kits supplied by Pharmacia Diagnostics AB (Upsala, Sweden). Gamma glutamyl transpeptidase (GGT) was measured at 37°C by the method of Haesen *et al.* (1972) using a Technicon II Auto-analyser. C-reactive protein (CRP) and alpha-l-acid glycoprotein (AGP) were measured by single radial immunodiffusion Mancini *et al.*, 1965) using anti-sera and standards obtained from Behringwerke, Marburg, Koln, Germany.

### *Statistical analysis*

A preliminary analysis of the relative frequency of positive responses to each GI question was performed using a $\chi^2$ test to detect significantly different response rates for cancer patients compared to the remaining groups. Similarly, the cumulative frequency distribution of each biochemical variable was plotted and, by using the 95th percentile value of the benign group as a cut-off point, the sensitivity and specificity of each marker to detect cancer were determined.

A logistic discriminant analysis (Anderson, 1972; Albert, 1982) has also been employed in this study to determine which variables are significant in discriminating between the cancer and non-cancer subjects. A stepwise procedure was adopted in which variables (4 tumour markers and 18 GI questions) are added to the model sequentially and at each step the statistical significance for each term not already in the model is calculated. The most significant variable at each step is added and when no variable is significant at the 5% level the process stops. Biochemical measurements underwent a logarithmic transformation (log 10) and positive responses to the questionnaire were accorded a score of +1 and a negative response −1. Sex was coded as +1 for male and −1 for female. The analysis was performed using the statistical package BMDP81, subroutine PLR, on the University of Leeds AMDAHL 470 computer.

To fit the model we used 54 cancer cases and the non-cancer group comprised 80 benign and 200 control population (first set data). As more cases were enrolled it was hoped that the model could be applied prospectively, thus permitting a more accurate impression of the validity of the model in a clinical setting (second set data).

### Results

The sensitivity and specificity for the individual biochemical markers using an arbitrary cut-off point equal to the 95th centile of the benign group are shown in Table II. Thus the single most sensitive agent was CRP with an overall detection for cancer of 52%.

$\chi^2$ analysis of the 18 GI questions revealed 6 questions which significantly distinguished between cancer and non-cancer subjects (Table III).

**Table II** Percentage of patients with a tumour marker value greater than the 95th centile of the benign group

| Tumour marker | Cut off value (95th centile benign) | Cancer (%) Colorectal | Gastric | Normal (%) |
|---|---|---|---|---|
| CEA | $>10\,ng\,ml^{-1}$ | 47.4 | 48.3 | 2.5 |
| AGP | $>1.4\,g\,l^{-1}$ | 36.8 | 65 | 2.6 |
| CRP | $>12\,mg\,l^{-1}$ | 4.4 | 69 | 2.8 |
| GGT | $>50\,U\,l^{-1}$ | 14 | 10 | 2.1 |

**Table III** Percentage frequency of positive responses per question for cancer and non-cancer groups

| | Cancer | Non-cancer[a] | P value ($\chi^2$ test) |
|---|---|---|---|
| Reduced appetite | 56.7 | 29.78 | 0.0012 |
| Weight loss | 64.7 | 30.7 | 0.0001 |
| Food sticking | 33.3 | 24.6 | 0.0001 |
| Nausea | 24.0 | 42.0 | 0.02 |
| Altered bowel frequency | 71.9 | 37.8 | 0.0001 |
| Altered stool appearance | 62.7 | 36.7 | 0.0025 |

[a]Non-cancer = Patients with benign disease and normal individuals.

However 35% non-cancer bearing subjects had 3 or more positive responses present.

We used the first set of data to fit a logistic model to discriminate the cancer from the non-cancer group. Using only the biochemical data, 36 (67%) of the 54 cancer patients were correctly classified, with a false positive rate of only 5%. The 18 cancers missed by this simple discriminant included 5 patients with liver metastases from colorectal cancer and 2 patients with advanced gastric cancer. A similar analysis of the 18 GI questions correctly classified 60% cancers with a 5% false positive rate. In a logistic analysis using both the questionnaire and biochemical data, 50 cancers (92%) were separated from the non-cancer groups, with a similar 5% false positive rate (Table IV). This is a significant improvement on both the questionnaire and biochemical data when used individually ($P<0.02$, $\chi^2$ test). The cancers misidentified were 2 colorectal cancers (Dukes' stage C+D) and 2 gastric cancers (stage II+IV).

**Table IV** Results of multivariate analyses applied to the initial data set

| Variables | Sensitivity ($n=54$) | Specificity (%) |
|---|---|---|
| Biochemical markers | 36 (67)[a] | 95 |
| Questionnaire | 33 (60) | 95 |
| Markers plus questionnaire | 50 (92) | 95 |

[a]Percentage in parenthesis.

The fitted model is determined by the discriminant function (log to base 10): $y=0.605$ (sex) $+0.112$ (age) $+2.73$ log (CRP) $+5.33$ log (CEA) $-4.09$ log (GGT) $+1.05$ (wt loss) $+0.968$ (bowel habit) +constant (8.4) and the probability of cancer is then $P=\exp(y)/(1+\exp(y))$.

The 'optimal' cut-off point for these values to indicate cancer is $P\geqq 0.275$.

By applying this criterion to the second set data, 28 of 32 cancers (88%) were selected but the specificity fell to 89%. The 4 cancers misclassified as low risk for cancer were all colorectal (Dukes' C).

Application of this type of analysis to the patients with benign disease, lead to 26 of the 168 individuals being identified as at 'high risk' of cancer. However, included in the 26 there were 4 subjects with gastric ulcer or polyps and 4 patients with large colonic adenomata and villous polyps. Thus the system detected further 'high-risk' potentially premalignant conditions which clinicians would wish to investigate.

Fifty-six subjects of the 720 individuals in the GP study were classified by the analyses to be at high-risk for cancer. Only seven were investigated but no neoplasia was detected. In the remainder, raised acute phase reactant proteins (APRPs) due to upper respiratory tract infections (the reason for the GP consultation) may have caused the high probability value. To date, with a follow up of 18

months, no cancers have been identified in these 56 subjects.

### Discussion

The problems of using a single tumour marker for cancer detection are again well demonstrated in this study. Even by taking the 95th percentile value of the patients with benign disease as the cut-off point for CEA detecting cancer, there is still 2.5% of the normal population with elevated CEA levels, whilst the sensitivity for cancer was only 47%. However, with a logistic analysis using the combination of four biochemical markers, we have confirmed the approach of de Mello *et al.* (1983) in that 36 (67%) cancers were detected in the first analysis, with a 5% false positive rate.

The analysis of the symptoms showed that 35% of the general population had at least three positive GI responses in the questionnaire. Eleven per cent had noticed rectal bleeding at some time (6% within a year) and 27% had experienced episodes of diarrhoea which somewhat dilutes these symptoms as potential markers for GI malignancy. Using the multivariate approach, only 60% cancers were correctly classified, thus showing the considerable overlap of symptoms between benign disease and cancer bearing subjects. This raises doubts concerning the validity of symptom complex analysis to determine cancer risk.

Combining the questionnaire data and the biochemical values, however, a significant improvement in the diagnosis of cancer has been achieved ($P<0.02$, $\chi^2$ test). By demanding a cut-off point which assured a high level of specificity in the first data set, we feel that we have managed to reduce the degree of false positivity that would normally be expected in a second phase study. Thus the recognition of 88% cancers with an 11% false positive rate in the second data set using this method is encouraging. It could be argued that by accepting a low level of probability of cancer ($P<0.275$) we have ensured a high sensitivity. However, by utilising a large number of controls we have shown that few 'normal' individuals would be selected for investigation despite the presence of multiple symptoms in 30% of the population. Furthermore, few of the benign disease group (15%) would be selected for investigation, implying that the cut off is satisfactory. The classification of patients with gastric ulcers and polyps and colorectal polyps into the high risk group for cancer may be seen not as a failure of the system but potentially useful to recognise a 'pre-malignant' condition and would fit the significant disease category of Mann *et al.* (1983).

The follow up of the 26 subjects with benign disease who were labelled as 'high risk' has revealed one cancer already. This individual with upper GI symptoms who had negative barium meal and gastroscopy, but re-presented six months later, was found to have a carcinoma of the fundus of the stomach. The probability of cancer at the first presentation was 80% using the questionnaire and tumour marker data. We await with interest the outcome of those normal subjects who had a high probability and were not investigated subsequently.

Such results in a preliminary study must always be met with caution. Whilst the number of non-cancer subjects used in this study is larger than previous reports, the whole study is still small and we would envisage a rather prolonged prospective collection of data before evaluating the potential of this approach. Further to miss six potentially curable colorectal cancers and one curable gastric cancer is not to be considered lightly. However, all three symptomatic Dukes' A and 13 of 14 Dukes' B colorectal cancers were detected. We would stress that the objective is to identify 'risk' for cancer and therefore to define priority for investigation when dealing with a symptomatic population. Any patient with persistent symptoms could easily be referred sooner irrespective of the probability value.

The basic cost for this approach must be considered as a balance between expenditure on these tests *versus* any reduction in investigation costs by reduced referral rates. The biochemical analyses cost £5.00 per head (including labour but excluding overheads), the brunt of which is the price of the CEA kits. This could be offset by the reduction in unnecessary investigations where the probability of cancer is extremely low.

In conclusion, we would suggest that neither biochemical variables or symptom analysis alone will define 'cancer risk' as accurately as the combination of both in a multivariate analysis. We would cautiously recommend further interest and recommend this approach as a possible way of using resources to identify those symptomatic patients who should be fully investigated for cancer.

### References

ALBERT, A. (1982). On the use of computation of likelihood ratios in clinical chemistry. *Clin Chem.*, **28**, 1113.

ANDERSON, J.A. (1972). Separate sample logistic discrimination. *Biometrika*, **59**, 19.

CHISHOLM, E.M., DEDOMBAL, F.T. & GILES, G.R. (1985). The validation of a self-administered questionnaire to elicit gastrointestinal symptoms. *Brit. Med. J.*, **290**, 1795.

CHU, C.Y-T., LAI, L.T-Y. & POKALA, H.P. (1982). Value of plasma Alpha-l-acid glycoprotein assay in the detection of human colorectal cancer: A comparison with carcinoembryonic antigen. *J. Natl Cancer Inst.*, **68,** 75.

DEMELLO, J., STRUTHERS, L., TURNER, R., COOPER, E.H., GILES, G.R. AND THE YORSHIRE REGIONAL GASTROINTESTINAL CANCER RESEARCH GROUP. (1983). Multivariate analyses as aids to diagnosis and assessment of prognosis in gastrointestinal cancer. *Br. J. Cancer*, **48,** 341.

HAESON, J.P., BERENDS, G.T. & ZONDAG, H.A. (1972). An automated method for the determination of serum gamma glutamyl transpeptidase. *Clin. Chim. Acta*, **37,** 463.

HOLLIDAY, H.W. & HARDCASTLE, J.D. (1979). Delay in diagnosis and treatment of symptomatic colorectal cancer. *Lancet*, **i,** 309.

JONES, I.S.C. (1976). An analysis of bowel habit, its significance in the diagnosis of carcinoma of the colon. *Am. J. Protocol.*, **27,** 45.

MACADAM, D.B. (1979). A study in general practice of the symptoms and delay patterns in the diagnosis of gastrointestinal cancer. *J.R. Coll. Gen. Pract.*, **29,** 723.

MANCINI, G., CARBONARA, A.O. & HEZEMANS, J.F. (1965). Immunochemical quantitation of antigens by single radial immunodiffusion. *Immunochemistry*, **2,** 335.

MANN, J., HOLDSTOCK, G., HARMAN, M., MACHIN, D. & LOEHRY, C.A. (1983). Scoring system to improve cost effectiveness of open access endoscopy. *Brit. Med. J.*, **287,** 937.

NATIONAL INSTITUTE OF HEALTH CONSENSUS REPORT. (1981). Carcinoembryonic Antigen: Its role as a marker in the management of cancer. *Brit. Med. J.*, **282,** 373.

RASHID, J.A., O'QUIGLEY, J., AXON, A.T.R. & COOPER, E.H. (1982). Plasma profiles and prognosis in gastric cancer. *Br. J. Cancer*, **35,** 390.

THOMSON, W.G. & HEATON, K.W. (1980). Functional bowel disorders in apparently healthy people. *Gastroenterology*, **79,** 283.

WALKER, C. & GRAY, B.N. (1983). Acute-Phase reactant proteins and carcinoembryonic antigen in cancer of the colon and rectum. *Cancer*, **52,** 150.

WARD, A.M., COOPER, E.H., TURNER, R., ANDERSON, J.A. & NEVILLE, A.M. (1977). Acute phase reactant protein profiles: An aid to monitoring large bowel cancer by CEA and serum enzymes. *Br. J. Cancer*, **35,** 170.